# Harmit Singh Malik

**DOI:** 10.1093/gbe/evac092

**Published:** 2022-07-02

**Authors:** Adri K Grow

**Affiliations:** Department of Biological Sciences, Smith College, Northampton, MA 01063, USA

We are continuing our biography section, featuring former Society for Molecular Biology & Evolution (SMBE) president, Harmit S. Malik. The following is based on a February 2022 interview with Harmit.

## How Did You become a Scientist?

Harmit grew up in the coastal city of Bombay (now called Mumbai) in India. Like many scientists, Harmit did not travel a “traditional” path into the biological sciences, but instead started studying chemical engineering at the Indian Institute of Technology, in Bombay. In the final years of his undergraduate studies, Harmit’s interests were piqued by the offering of a new course entitled “Introduction to Molecular Biology.” Despite his best efforts to join the class, and even after attending the first few meetings, Harmit’s required chemical engineering courses clashed with the schedule. Although Harmit was captivated by the content in those very first lectures, he went to the professor to express his regrets that he could no longer participate in the class.

To Harmit’s surprise, Professor K. K. Rao offered to teach him the molecular biology course one-on-one in the afternoons as long as Harmit kept up with the readings. Throughout their sessions, Harmit could not help but realize the connections between engineering and biology, especially in light of evolution’s intricate systems that sense and adapt to the environment. At that same time, Harmit was also reading *The Selfish Gene* by Richard Dawkins and pinpointed contradictory concepts in biology. For example, on one hand, there is a beautifully orchestrated interaction between cells and their environment, but on the other hand, elements are running amok in the genome even at the expense of causing harm to the very genome they belong to. When Harmit brought these puzzling questions to Dr. Rao, he suggested Harmit devote a PhD to the topic. Harmit’s immediate thought was that he had no business even considering the idea given his academic background.

With the support of Dr. Rao, Harmit applied to graduate programs in the US with the intention of studying the selfish transposable elements that jump around the genome. At the University of Rochester, Harmit completed a PhD with advisor Dr. Thomas Eickbush studying retrotransposable elements. Harmit went on to be a postdoctoral researcher in Dr. Steven Henikoff’s lab at the Fred Hutchinson Cancer Research Center where he studied the evolution of centromeres and centromeric proteins. Today, Harmit is a professor of Basic Sciences and a Howard Hughes Medical Institute Investigator at the Fred Hutch running a lab that continues to investigate what sparked it all for him, the selfish genes and genetic conflicts that make up the complex workings of the genome.

## Does Your Engineering Background Influence Your Biological Research?

In some senses, Harmit finds the engineering background helpful, but not necessarily in the way you might think. His background in engineering allows him to break down problems quickly and start from the small pieces at the bottom that seem trivial as opposed to the top-down view common among traditionally trained biologists. Harmit enjoys the continuous process of learning. Undergraduates will say something in passing about a protein that completely catches Harmit off guard and that sparks an exciting conversation. Harmit used to try to hide his ignorance about information that others took for granted but realizes that knowing everything in a particular field is an unrealistic expectation and there are always opportunities to gather new insights.

## What Are Some Challenges You Have Faced in Your Career?

One of Harmit’s biggest challenges was having very little biology background, compounded with the fact that he had to teach undergraduate courses while taking PhD courses in the very subject. Of course, in the beginning, moving from tropical Bombay to upstate New York was a shock, and bouts of homesickness were common, but the university atmosphere was quite open-minded and there were many other international students. Over the years, some professors had a problem with Harmit wearing a turban in class, but overall he says that he has been lucky with his experiences in academics and attributes that to the fact that he’s lived in more liberal, cosmopolitan regions of the US. Harmit is keen to promote inclusion in science and is active on social media encouraging people to get involved in science and breaking down the barriers to entry into science.

## Your Favorite Contribution to the Literature?

A paper that Harmit is especially proud of is “The age and evolution of non-LTR retrotransposable elements” published in *Molecular Biology and Evolution* in 1999. This publication was the focal point of Harmit’s PhD thesis, which implemented a phylogenetic framework to synthesize retrotransposable element evolution. Using this framework, Harmit highlighted the gain and loss of enzymatic domains in retrotransposable elements in lineages from different parts of the tree of life. This work led to precise predictions of novel enzymatic domains, which were shown to be correct by other graduate students in the lab. Finally, this broad synthesis also provided evidence that some transposable elements can be transmitted vertically for long evolutionary periods, something that previously had lacked supporting data in the literature.

## What Do You Do for Fun Outside of the Sciences?

Outside of science, Harmit enjoys reading murder mystery novels including everything from Agatha Christie to the recently popular Nordic psycho thrillers. Like many of us who explored new hobbies during the pandemic, Harmit started reading more non-fiction books about science, policy, and the environment and if given a weekend off, he would spend it enjoying a really great book.

## What’s Some Advice for People Entering the Field of Science?

Often people are told to find their passion, but Harmit feels that his passion found him quite by accident, and not at a particularly convenient time, as he was all set to start a career in chemical engineering. However, new doors opened that allowed him to find supportive mentors as he navigated a transition between careers. From the perspective of a mentor to many, Harmit reminds young scientists that they do not owe their mentor anything beyond their own success and happiness, and oftentimes mentors find the most joy from participating at a critical juncture in their training.

## Your Final Thoughts?

Harmit believes that now is a really exciting time to work in the field of genomics and molecular evolution. Molecular biology is in an age where the tremendous amount of data intersects with many disciplines (e.g. healthcare, computer science), and as a result, Harmit warns that more attention needs to be given to ethical considerations of data. It is the obligation of researchers to know where their data comes from and how to use it responsibly. To wrap up our conversation, Harmit shares that employment opportunities in biology are becoming more diverse as the skills acquired during graduate school have broad applicability and are sought after outside the traditional academic landscape.

**Figure:**
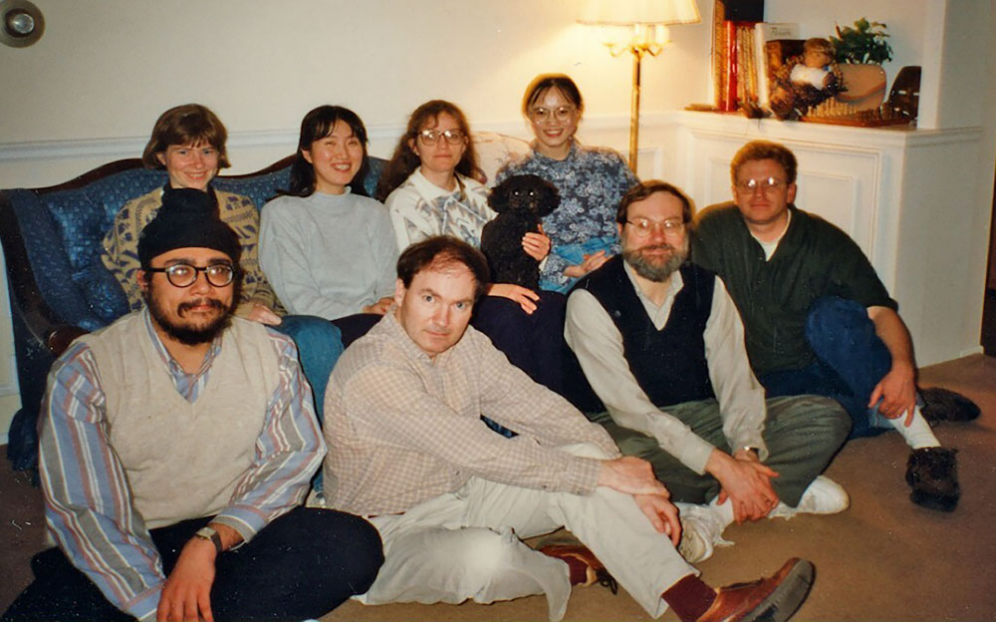
Harmit during graduate school among members of the Eickbush lab (1995). Back row (left to right): Janet George, Dongmei Luan, Danna Eickbush (and Coco), Jin Yang Front row (left to right): Harmit Malik, Bill Burke, Tom Eickbush, Warren (Trey) Lathe III

**Figure:**
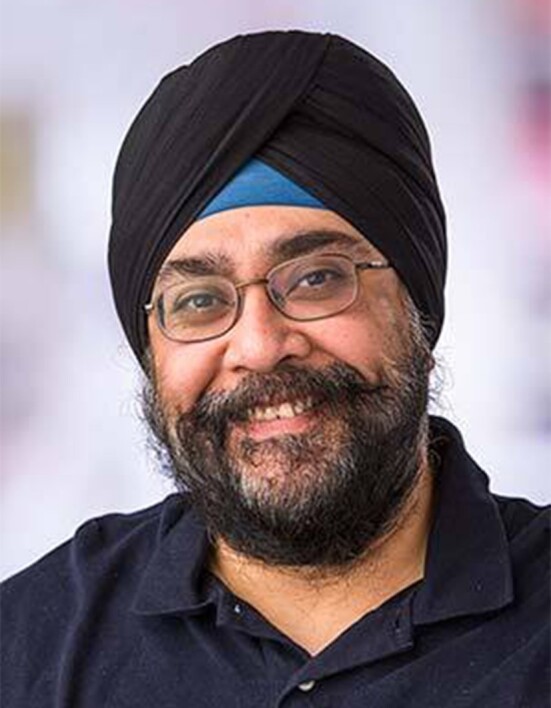
Harmit today as a professor of Basic Sciences and a Howard Hughes Medical Institute Investigator at the Fred Hutchinson Cancer Research Center.

